# Global expression profile in low grade meningiomas and schwannomas shows upregulation of *PDGFD*, *CDH1* and *SLIT2* compared to their healthy tissue

**DOI:** 10.3892/or.2014.3526

**Published:** 2014-10-03

**Authors:** MIGUEL TORRES-MARTIN, LUIS LASSALETTA, ALBERTO ISLA, JOSE M. DE CAMPOS, GIOVANNY R. PINTO, ROMMEL R. BURBANO, JAVIER S. CASTRESANA, BARBARA MELENDEZ, JUAN A. REY

**Affiliations:** 1 Molecular Neuro-Oncogenetics Laboratory, Research Unit, Hospital Universitario La Paz, IdiPAZ, Madrid, Spain; 2 Department of Otolaryngology, Hospital Universitario La Paz, IdiPAZ, Madrid, Spain; 3 Department of Neurosurgery, Hospital Universitario La Paz, IdiPAZ, Madrid, Spain; 4 Department of Neurosurgery, Fundacion Jimenez Diaz, Madrid, Spain; 5 Genetics and Molecular Biology Laboratory, Federal University of Piau, Parnaiba, Brazil; 6 Human Cytogenetics Laboratory, Institute of Biological Sciences, Federal University of Para, Belem, Brazil; 7 Department of Biochemistry and Genetics, University of Navarra School of Sciences, Pamplona, Spain; 8 Molecular Pathology Research Unit, Virgen de la Salud Hospital, Toledo, Spain

**Keywords:** schwannoma, meningioma, microarray, comparative gene expression, *NF2*, neurofibromatosis 2

## Abstract

Schwannomas and grade I meningiomas are non-metastatic neoplasms that share the common mutation of gene *NF2*. They usually appear in neurofibromatosis type 2 patients. Currently, there is no drug treatment available for both tumors, thus the use of wide expression technologies is crucial to identify therapeutic targets. Affymetrix Human Gene 1.0 ST was used to test global gene expression in 22 meningiomas, 31 schwannomas and, as non-tumoral controls, 3 healthy meningeal tissues, 8 non-tumoral nerves and 1 primary Schwann cell culture. A non-stringent P-value cut-off and fold change were used to establish deregulated genes. We identified a subset of genes that were upregulated in meningiomas and schwannomas when compared to their respectively healthy tissues, including *PDGFD*, *CDH1* and *SLIT2*. Thus, these genes should be thoroughly studied as targets in a possible combined treatment.

## Introduction

Schwannomas are benign tumors that arise from Schwann cells. They typically appear in the vestibulocochlear nerve and are considered to be grade I tumors; approximately 95% are unilateral and present sporadically, whereas 5% are associated with neurofibromatosis type 2 syndrome (NF2). Patients with NF2 present with bilateral schwannomas and other tumors, frequently meningiomas, which originate from arachnoid cells, and account for 20% of all primary intracranial tumors. The current classification of meningiomas by the World Health Organization (WHO) includes three grades: 90% are classified as grade I tumors; ~8–9% are atypical grade II tumors; and 1–2% are anaplastic/malignant grade III tumors (1). Meningiomas have a recurrence rate of 18, 40 and 80% for grade I, II and III, respectively.

Preliminary cytogenetic studies have demonstrated the absence of one chromosome 22 in both neoplasms (2,3), thus suggesting a common genetic origin for at least some subgroups of these neurogenic tumors. Subsequently, *NF2* gene (located at 22q12.2) inactivation was found to be due to several mechanisms, such as mutations or allelic loss due to monosomy or deletion of chromosome 22, accounting for up to 66% in schwannomas (4) and 18–50% in sporadic meningiomas, depending on the histopathological subtypes (5). In addition to the characteristic chromosome 22 loss, secondary alterations such as 1p deletions have been described in both tumor types, and these alterations appear to be related to tumor progression in meningiomas (6–8). Although DNA methylation studies on these neurogenic tumors have revealed the non-random involvement of this mechanism in the inactivation of some tumor-related genes (9–11), controversial data are available concerning the epigenetic (through CpG island aberrant methylation) *NF2* inactivation in both neoplasms (12–16). Indeed, recent studies on genome-wide methylation suggest that this mechanism is associated with malignant transformation in meningiomas, and allows for the epigenetic subclassification of this tumor (17,18).

Global exome sequencing in meningiomas showed that, in grade I tumors, *NF2* gene alteration (by mutation and/or loss of chromosome 22) is mutually exclusive with other gene mutations such as *AKT1, TRAF7, KLF4* and *SMO* (19), but not with others such as *NF1* and *NEGR1* (20). In schwannomas, no alternative mutation has been found for those samples lacking hits over *NF2* and, however, Merlin (the NF2 protein) does not seem to be present in the cases analyzed to date (21).

The expression analysis of tumor-related genes in meningiomas and schwannomas suggests a possible molecular subgroup classification in both tumors (22) with the involvement of differential regulatory pathways (23,24) related to the allelic losses at 1p and 14q in meningiomas (25). Whole genome expression analysis has been performed on schwannomas (26–28) and meningiomas (29–32). Whereas meningiomas have shown differential expression patterns based on progression and recurrence, but not strictly supported by grade (31), in schwannomas no distinctive pattern has been found using clinical correlations (28). However, a critical deregulation of microRNAs, including the upregulation of those located at the 14q32 chromosomal region, was a characteristic feature of vestibular tumors (33).

Intracranial non-recurrent WHO grade I meningiomas and schwannomas represent similar problems for patients, depending on the brain structures affected by their non-invasive growth. Currently, treatment options for patients with grade I meningiomas or schwannomas include surgery resection, radio-surgery and a ‘wait and see’ strategy. Thus, there is no available chemotherapeutic treatment for these tumors besides surgery, a situation especially traumatic for patients suffering bilateral vestibular schwannomas and several meningiomas such as those affected by NF2. Due to the common genetic origin of these tumors (*NF2* inactivation), previous studies have attempted to identify targets with which to inhibit both schwannoma and meningioma progression. AR42, a histone deacetylase inhibitor, was found to suppress the proliferation of meningioma and schwannoma cell lines *in vitro* (34), and the same effect was shown by cucurbitacin D and goyazensolide in primary cultures (35).

In the present study, we used microarray technology to compare gene-expression patterns and identify genes and pathways of potential interest as key targets for the combined treatment of vestibular schwannomas and grade I meningiomas.

## Materials and methods

### Statementofethicsandsamples

The local Ethics Review Board of La Paz University Hospital approved the study protocol according to the principles of the Declaration of Helsinki. All patients received detailed information concerning the study and provided their written informed consent prior to their inclusion. In this study, we used RNA from 22 meningiomas, 31 schwannomas and, as non-tumoral controls, 3 healthy meningeal tissues, 8 non-tumoral nerves and 1 primary Schwann cell culture. The three control non-tumoral meningeal RNAs derived from two healthy males and one female and were purchased from BioChain^®^ (cat. no. R1234043-10-D03; lot nos. B108134, A602330 and B501146).

### RNA extraction and microarray experiments

The RNA was extracted with the RNeasy^®^ Mini kit (Qiagen, Valencia, CA, USA) as indicated previously (28). For global gene expression, the Affymetrix Human Gene 1.0 ST was used. The expression profile of the meningiomas and the meninges samples can be accessed at the gene expression Omnibus (GEO) database GSE54934. The arrays of schwannomas and control nerves were previously published (28) and are available at the GEO database GSE39645. The arrays were processed at the Institute for Research in Biomedicine (IRB), Barcelona, Spain.

### Statistical analysis

The normalization and summarization were performed using the robust multichip average (RMA). In order to reduce the batch effect among tumors (schwannomas and meningiomas) and controls (healthy nerves and meninges), a critical aspect for our analysis, we used ComBat (36). For data analysis, the genes were considered deregulated between groups when at least a 2-fold change in expression and a P<0.05 cut-off (ANOVA) was identified, as previously recommended by the MAQC consortium (37). For the comparison between schwannomas and meningiomas in order to obtain a list of genes with no changes among both tumor types, we used a more restrictive fold (<1.5) exclusively for this purpose, since the ComBat effect could have lowered these values and false-positives could appear. For comparative purposes, a list of differentially expressed genes and fold-change was obtained with the Geo2R web tool (http://www.ncbi.nlm.nih.gov/geo/geo2r/) in the series GSE43290, which includes 4 meninges as controls and 47 tumors (29). As our meningioma series mainly included grade I tumors, only the 33 WHO grade I meningiomas and the 4 controls included in this report were used for comparison.

### DNA extraction

The DNA was extracted by standard methods, as previously described (22). The data regarding the *NF2* status, including loss of heterozygosity of 22q (LOH), Multiplex ligation-dependent probe amplification (MLPA) of *NF2* (SALSA P044) and sequence analysis by dHPLC were reported previously in detail (22,28), and were performed as described (22). Clinical and *NF2* status data from the meningiomas correspond to cases M02, M04, M05, M07, M09, M10, M12, M14, M24, M25, M28, M29, M30, M31, M32, M33, M34, M38, M39, M40, M41 and M42, as previously reported (22). The complete case series of schwannomas from our previous report (28) was included.

## Results and Discussion

### Comparison with respect to previous analyses of meningiomas and the summary of results in schwannomas

Meningioma profiling was analyzed extensively in previous studies, and up to five expression subgroups were characterized (31), although this classification did not represent the actual WHO classification. Recurrence and progression appear to play a relevant role in the expression pattern of these tumors (32), and two meningioma groups were identified showing different clinical and pathological behaviors, more related to clinical outcome than to WHO grade *per se*. Furthermore, depending on the cytogenetic aberrations, differential expression patterns have been described (25,29). Tumors that presented monosomy of chromosome 22 and cases with multiple karyotype alterations had a differential expression pattern, whereas those cases with deletion of chromosome 1 alone showed random behavior (29). In summary, previous analyses of gene-expression patterns in meningiomas do not seem to accurately represent the current WHO classification, although recurrence and progression status might be reflected in these studies. We used 20 grade I meningiomas, 2 grade II meningiomas and 3 healthy meninges. Practically the same values were obtained when meningiomas grade II were removed from the study (data not shown). When we compared our results in meningiomas with those obtained from the dataset GSE43290 (29), we found a high consistency in our results, such as the downregulation of diverse genes such as *SNAP25, MBP, TTR* and *VSNL1*, and the upregulation of *FBN2, FGF9* and *SULF1* (full data available upon request). As in previous studies, our 2 cases of meningioma grade II did not show a different trend. The schwannoma expression profile was previously explained (28). In brief, the upregulation of *SPP1, MET* and associated genes or *LATS2* was reported, whereas the downregulation of *CAV1, AR* and *PAWR* was found. In general, myelinization genes were overexpressed, suggesting that schwannoma cells could resemble a previous state of mature Schwann cells.

### Gene co-overexpression in meningiomas and schwannomas

Using the ANOVA test at P<0.05 significance across the four groups (all meningiomas, schwannomas, control healthy meninges and control nerves), we obtained a list of 12,395 genes with differential expression among these four groups. Of those, 346 (data not shown; available upon request) did not meet the criteria established for accepting deregulation differences between both tumor groups, which was ≤1.5-fold of the differential expression between the schwannomas and the meningiomas, a limit value selected as deregulated between these two groups due to the correction effect of ComBat. Among those 346 genes with similar expression in tumors, 47 showed co-overexpression in schwannomas and meningiomas when compared with their respective controls at 2-fold (as ComBat correction would only be based on batch effect) (Table I). These genes included E-cadherin (*CDH1*), which is usually silenced by several mechanisms, such as the Wnt signaling pathway in human cancer, including meningiomas (38), and platelet-derived growth factor D (*PDGFD*), an activator for PDGFR-β (39). This pathway has been reported as overexpressed in multiple cancer types such as pancreatic cancer and brain tumors, including schwannomas (39). Another gene reported as expressed (and protein present) in meningiomas and schwannomas is tyrosine kinase receptor *MET* (40), which is responsible for cell migration, anchorage-independent growth and many other functions. High levels of this receptor have been found in a wide variety of tumors, such as breast cancer, renal cell carcinoma and head and neck tumors (41). Mechanisms such as point mutations, alternative splicing, genomic amplification and transcript amplification appear to participate in overexpression of *c-MET* (reviewed in ref. 42). Accordingly, we found *MET* upregulation in both neoplasms compared with their respective control tissues, and again, a similar level of expression between both tumor types was detected. *SLIT2* is a member of the Slit family that modulates cell migration by binding with the Robo family. This gene has been found expressed in the development of several malignancies such as colorectal epithelial cell carcinogenesis (43). The findings in this report (43), suggest that Slit2-Robo1 causes E-cadherin degradation, and although our results show an upregulation of the E-cadherin gene, the former mechanism should not be ruled out in the tumors we studied. In other neoplasms, although expressed, *SLIT2* does not seem to play any role (44). In agreement with the data from the study selected for validation (29), several genes, including *CDH1, PDGFD, CX3CR1, CCND1* and *SLIT2*, were also upregulated, as shown in data obtained from meningioma dataset GSE43290 (29); in contrast, *MET* showed a trend of upregulation but did not reach 2-fold. Functional annotation using DAVID showed enrichment in inflammatory response, cell migration and defense response (data not shown; available upon request).

### Gene co-infraexpression in meningiomas and schwannomas

A total of 35 genes (Table II) with no difference in expression between schwannomas and meningiomas were underexpressed when compared with their respective controls in both neoplasms. Among them are selectin E (*SELE*) and Rho family GTPase 1 (*RND1*), which is linked to semaphorins (45,46) and cytoskeleton organization in axons. The chemokine (C-X-C motif) ligand 2 (*CXCL2*) was significantly downregulated in schwannomas and meningiomas, whereas the opposite trend has been shown in malignant neoplasms such as ovarian and endometrial cancer and oral squamous cell carcinoma (47). As schwannomas and meningiomas are usually non-invasive, this fact could explain the different trend in deregulation of *CXCL2*. Stathmin-like 2 (*STMN2*) showed the same pattern: upregulation in hepatoma cells but downregulation in schwannomas and meningiomas. Notably, *STMN2* interacts with Rho family GTPase 1 (*RND1*) in axon extension (48), another gene that was downregulated in both tumors. Other downregulated genes in both tumors were E-selectin (*SELE*) and vascular adhesion protein 1 (*AOC3*), related to the tethering and rolling of leukocytes (49); thus, the non-invasive nature of grade I meningiomas and schwannomas could explain the downregulation of these genes. Validation with the dataset GSE43290 was performed, and included, among others, downregulation of *AOC3, STMN2, SELE, RGS4, THBS4* and *RND1*. Functional analysis with DAVID included leukocyte and cell migration, heparin binding or membrane fraction (data available upon request).

### Gene expression differences between meningiomas and schwannomas

The main goal of our study was to test gene expression profiles common to schwannomas and meningiomas in regard to their respective controls, and having taken into account their relative expression. However, we also studied the gene expression differences between both neurogenic neoplasms. As samples were processed in various batches, we used a Bayesian method to reduce the batch effect. Because of this effect, the differential expression of certain genes in schwannomas and meningiomas could have decreased. This issue, although it limits our information, is vital to our study since the batch effect was very marked; 192 genes were upregulated at 1.5-fold differences and P<0.05 (data available upon request) in schwannomas as compared to meningioma expression. Most of these genes are related to neuron migration and the myelin sheath, such as the following: peripheral myelin protein 2 (*PMP2*), expressed in the cytoplasmic side of myelin in the peripheral nervous system (50); myelin protein zero (*MPZ*), representing 50% of the total myelin protein in the peripheral nervous system (51); neurexin 1 (*NRXN1*), which mediates formation and maintenance of synaptic junctions (52); and neural cell adhesion molecule 2 (*NCAM2*), which is involved in axonal projection (53).

Upregulation in meningiomas compared with schwannomas gave us 88 genes (data available upon request) and included cellular retinoic acid binding protein 2 (*CRABP2*), a chaperon downregulated in high-grade gliomas (54), and secreted frizzled-related protein 2 gene (*SFRP2*), a gene identified as a tumor suppressor in a renal cell carcinoma cell line (55).

Another comparison concerned those genes that were upregulated in schwannomas with respect to nerves, and downregulated in meningiomas with respect to healthy meninges. These findings included genes such as hepatocyte cell adhesion molecule (*HEPACAM*), neuritin 1 (*NRN1*) and kinesin family member 1A (*KIF1A*).

**Figure 1:**
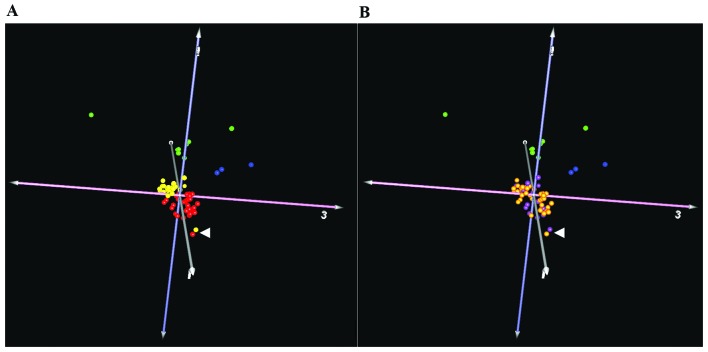
Principal component analysis (PCA) of all samples studied. Green dots represent non-tumoral healthy nerves and the blue dots non-tumoral meninges samples. (A) Red dots are all the schwannomas studied, while yellow dots are the meningioma samples. Both entities are clearly separated, except for two samples (white arrow), one corresponding to a schwannoma from an NF2 patient and the other to a grade I meningioma. The most remote green dot corresponds to the cultured Schwann cells used as controls. (B) Violet dots are samples without alteration in the *NF2* gene, and gold dots are tumors carrying such an alteration. The same point of view is shown in both images A and B, and no clear distinction between those two groups was found.

The *NF2* mutation rate (determined by sequencing, MLPA and chromosome 22q LOH analyses) in this series was 74% for schwannomas and 68% for meningiomas. We compared the expression patterns in samples from both tumor types, and with respect to the presence or lack of any alteration in the *NF2* gene (38 samples with alteration and 15 without any). Using these groups, we identified 2 genes with differential expression levels. The natriuretic peptide receptor C/guanylate cyclase C (atrionatriuretic peptide receptor C) (*NPR3*) was downregulated in those samples without *NF2* alterations. This gene codes for a receptor coupled to various signaling transduction cascades in several tissues such as cardiac myocytes and fibroblasts (56). On the other hand, the G antigen 12J (*GAGE12J*) gene, transcribed in human fetal and tumoral tissues (57), was also downregulated, but on this occasion in tumors with *NF2* alteration. As only 2 genes were detected, based on our microarray results in both neoplasms, it would seem that there is no differentiated subset of expression profiles of genes between samples with or without alteration over *NF2* in grade I meningiomas and schwannomas (Fig. 1). Nevertheless, single genes could be altered in tumors with or without *NF2* alteration, although such a reduced number could be due to outlier values.

At present, there is no chemotherapeutic treatment available for either meningiomas or schwannomas, thus research for a combined solution could be of great value to those patients affected with both tumor types, primarily patients with neurofibromatosis type 2. In this study, we found a set of genes with aberrant expression in both entities compared with their respective control tissue, but with similar expression levels between these tumors, including PDGF, c-Met or Slit2 pathways. Thus, these and the other genes identified in this study, and their regulatory pathways, might be of interest for further experiments in the search for common solutions for patients affected by schwannomas and meningiomas.

## Figures and Tables

**Table I:** Genes overexpressed in meningioma and schwannoma when compared with their respective control tissue.

Gene	Database	Chromosome	C-M	N-S	M-S	P-value
*CDH1*	NM_004360	16q22.1	5.4	5.8	−1.0	8.35E-09
*PDGFD*	NM_025208	11q22.3	4.4	6.2	−1.1	7.48E-13
*SLIT2*	NM_004787	4p15.2	3.6	5.8	−1.1	2.02E-13
*HLA-DPA1*	NM_033554	6p21.3	2.9	3.9	−1.1	5.24E-07
*PAPPA*	NM_002581	9q33.2	2.8	3.8	−1.1	3.68E-06
*TREM2*	NM_018965	6p21.1	2.7	5.5	−1.3	2.8E-12
*HLA-DPA1*	NM_033554	6p21.3	2.6	4.1	−1.2	5.24E-07
*HPGDS*	NM_014485	4q22.3	2.6	2.4	−1.1	3.83E-07
*GPR34*	NM_001097579	Xp11.4	2.6	12.0	−1.5	9.9E-11
*CX3CR1*	NM_001337	3p21|3p21.3	2.5	5.7	−1.2	2.15E-07
*ANKRD22*	NM_144590	10q23.31	2.5	7.3	−1.4	1.04E-06
*C3*	NM_000064	19p13.3-p13.2	2.4	2.6	−1.2	5.77E-05
*CYBB*	NM_000397	Xp21.1	2.4	4.1	−1.2	1.11E-07
*LGALS3BP*	NM_005567	17q25	2.4	2.9	−1.1	4.67E-13
*WIPI1*	NM_017983	17q24.2	2.4	2.0	−1.0	2.55E-12
*APOBEC3C*	NM_014508	22q13.1	2.4	2.5	−1.1	3.35E-08
*C3AR1*	NM_004054	12p13.31	2.4	4.8	−1.2	4.43E-09
*FCGBP*	NM_003890	19q13.1	2.3	9.7	−1.3	1.03E-11
*FRAS1*	NM_025074	4q21.21	2.3	3.2	−1.2	7.42E-08
*FLRT3*	NM_198391	20p11	2.3	3.8	−1.2	1.76E-05
*FCGR1A*	NM_000566	1q21.2–q21.3	2.3	4.3	−1.3	1.66E-08
*MET*	NM_001127500	7q31	2.3	4.9	−1.3	1.22E-08
*ITPR3*	NM_002224	6p21	2.3	3.7	−1.1	3.69E-12
*FCGR1B*	NM_001017986	1p11.2	2.3	2.9	−1.2	4.14E-08
*ALCAM*	NM_001627	3q13.1	2.2	2.5	−1.0	4.26E-09
*HLA-DPB1*	NM_002121	6p21.3	2.2	4.6	−1.3	1.75E-07
*LAMB1*	NM_002291	7q22	2.2	2.0	−1.2	4.57E-07
*C8orf84*	NM_153225	8q21.11	2.2	2.6	−1.2	8.11E-06
*SLFN12*	NM_018042	17q12	2.2	2.3	−1.2	4.14E-10
*FCGR1A*	NM_000566	1q21.2–q21.3	2.2	3.1	−1.2	1.66E-08
*LHFPL2*	NM_005779	5q14.1	2.1	2.3	−1.1	8.13E-09
*MS4A6A*	NM_152852	11q12.1	2.1	4.5	−1.3	4.8E-08
*CD84*	NM_001184879	1q24	2.1	2.7	−1.2	3.38E-09
*TRIM22*	NM_006074	11p15	2.1	2.2	−1.1	2.09E-09
*CD4*	NM_000616	12pter-p12	2.1	2.5	−1.1	1.69E-07
*CSF1R*	NM_005211	5q32	2.1	3.8	−1.2	5.21E-08
*GFRA1*	NM_005264	10q26.11	2.1	4.9	−1.4	1.34E-07
*HLA-DPB1*	NM_002121	6p21.3	2.1	4.5	−1.3	1.75E-07
*CD86*	NM_175862	3q21	2.1	2.9	−1.3	1.03E-06
*C1QA*	NM_015991	1p36.12	2.1	4.3	−1.2	1.24E-07
*TLR7*	NM_016562	Xp22.3	2.0	3.6	−1.3	7E-08
*CCND1*	NM_053056	11q13	2.0	2.7	−1.1	1.48E-11
*HLA-DQA1*	NM_002122	6p21.3	2.0	2.6	−1.2	4.19E-05
*FAM105A*	NM_019018	5p15.2	2.0	2.6	−1.1	9.2E-08
*C6orf138*	NM_001013732	6p12.3	2.0	3.4	−1.2	1.99E-10
*P2RY13*	NM_176894	3q24	2.0	2.4	−1.2	3.08E-06
*PROS1*	NM_000313	3q11.2	2.0	5.0	−1.4	9.1E-14

Official gene symbol is shown for every gene. C-M values correspond to the fold-change value of control healthy meninges (C) minus meningioma (M). In the case of N-S, N refers to nerve healthy tissue minus schwannoma (S). In the column M-S, meningioma (M) minus schwannoma (S) is performed. Only those genes with ≤1.5 fold-change between both tumors are shown.

**Table II:** Genes infraexpressed in meningioma and schwannoma when compared with their respective control tissue.

Gene	Database	Chromosome	C-M	N-S	M-S	P-value
*SAA1*	NM_000331	11p15.1	−2.0	−2.7	1.1	0.000414
*INHBA*	NM_002192	7p15-p13	−2.0	−4.1	1.2	2.42E-09
*PCDH18*	NM_019035	4q31	−2.0	−2.6	1.2	0.000183
*PTGIS*	NM_000961	20q13.13	−2.1	−3.4	1.3	1.72E-06
*HHIP*	NM_022475	4q28–q32	−2.1	−2.9	1.1	1.44E-06
*AQP9*	NM_020980	15q	−2.1	−4.5	1.0	2.78E-08
*TCEAL2*	NM_080390	Xq22.1–q22.3	−2.1	−2.4	1.4	0.000135
*S100A12*	NM_005621	1q21	−2.2	−5.6	1.1	9.51E-07
*PDE3A*	NM_000921	12p12	−2.2	−2.0	1.2	2.03E-08
*S100A9*	NM_002965	1q21	−2.2	−4.6	−1.0	2.63E-05
*PAK3*	NM_002578	Xq23	−2.3	−4.1	1.3	5.36E-11
*SLC16A7*	NM_004731	12q13	−2.3	−2.2	1.1	4.89E-12
*PI16*	NM_153370	6p21.2	−2.3	−5.1	1.2	1.76E-09
*MGST1*	NM_145792	12p12.3-p12.1	−2.3	−3.6	1.1	0.001079
*FGFR2*	NM_000141	10q26	−2.3	−2.6	1.3	1.69E-09
*TRPM3*	NM_206946	9q21.12	−2.4	−2.0	1.1	3.65E-11
*PDZRN4*	NM_013377	12q12	−2.5	−3.0	1.1	1.14E-08
*THBS4*	NM_003248	5q13	−2.5	−3.5	1.0	1.71E-09
*STEAP4*	NM_024636	7q21.12	−2.7	−4.2	1.1	1.38E-07
*DCLK1*	NM_004734	13q13	−2.8	−2.4	1.1	2.22E-07
*ZNF385D*	NM_024697	3p24.3	−3.0	−2.0	1.1	1.34E-05
*CXCL2*	NM_002089	4q21	−3.1	−3.3	−1.0	7.52E-09
*FABP4*	NM_001442	8q21	−3.1	−13.5	1.2	1.29E-12
*IL6*	NM_000600	7p21	−3.1	−4.6	−1.0	0.000397
*SELE*	NM_000450	1q22–q25	−3.3	−6.6	1.0	2.29E-09
*SLC14A1*	NM_001128588	18q11–q12	−4.0	−3.3	1.0	2.7E-09
*APLNR*	NM_005161	11q12	−4.8	−3.1	−1.0	8.05E-11
*ADH1B*	NM_000668	4q23	−4.9	−3.6	−1.1	6.95E-07
*ADCYAP1R1*	NM_001118	7p14	−5.1	−3.3	1.0	6.98E-11
*RND1*	NM_014470	12q12	−5.4	−3.1	−1.0	3.5E-09
*ADAMTS1*	NM_006988	21q21.2	−6.0	−2.4	−1.1	4.64E-11
*HSPB8*	NM_014365	12q24.23	−6.0	−3.5	1.0	5.3E-07
*AOC3*	NM_003734	17q21	−6.3	−2.5	−1.1	3.64E-11
*RGS4*	NM_001102445	1q23.3	−6.7	−2.1	−1.1	2.55E-10
*STMN2*	NM_007029	8q21.13	−9.2	−2.9	−1.1	4.92E-11

Official gene symbol is shown for every gene. C-M values correspond to the fold-change value of control healthy meninges (C) minus meningioma (M). In the case of N-S, N refers to nerve healthy tissue minus schwannoma (S). In the column M-S, meningioma (M) minus schwannoma (S) is performed. Only those genes with ≤1.5 fold-change between both tumors are shown.
